# Ophthalmological complications related to the use of microfocused ultrasound in the periocular region and face^⋆^

**DOI:** 10.1016/j.abd.2021.12.013

**Published:** 2023-06-23

**Authors:** Mariana Marques Rechuan, Jordana Cezar Vaz, Fernanda de Azevedo Pereira Marques, David Rubem Azulay

**Affiliations:** Instituto de Dermatologia Professor Rubem David Azulay, Santa Casa da Misericórdia do Rio de Janeiro, Rio de Janeiro, RJ, Brazil

Dear Editor,

The authors describe a severe clinical case of ophthalmologic complications related to the use of microfocused ultrasound (MU) in the periocular region and face. It is extremely important to know about this possible complication of the use of MU.

A 50-year-old female patient sought dermatological care for facial rejuvenation. After evaluation, MU was employed on the face and periocular region, with carboxytherapy being performed on the eyelids. There were no complications during the procedures, which were followed by the application of Cicaplast® cream on the palpebral region. Immediately after the procedure, she complained of visual blurring, initially attributed to the use of Cicaplast®. On the first and second days after the procedure, she developed edema and erythema limited to the orbicularis region, while the visual blurring persisted and she started to have scotomas, lacrimation, and, finally, eye pain. On the third day, she experienced worsening of the ophthalmological condition, making it impossible to perform her daily activities, due to visual blurring and pain.

She was assessed by an ophthalmologist who found a significant increase in eye pressure, iris retraction, areas of fibrosis (Fig. 1), and crystalline lens atrophy in the shape of spicules, and she was diagnosed with acute angle-closure glaucoma and cataracts in the shape of spicules.

**Figure 1:**
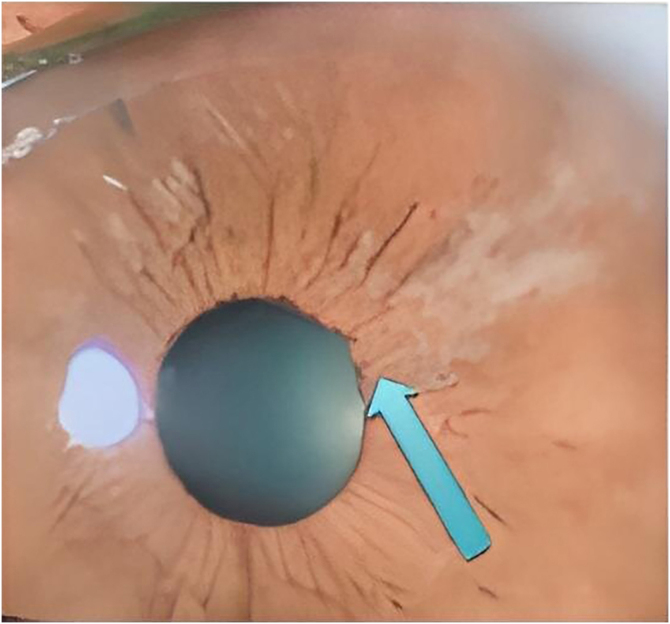
Patient’s ophthalmoscopy, showing iris retraction and areas of fibrosis

Treatment was implemented with maximum doses of timolol, brimonidine, bimatoprost, and acetazolamide eye drops; however, there was some difficulty in reducing ocular pressure and little improvement of symptoms. Surgical intervention was then considered to preserve the optic nerve. After six months of treatment, she showed partial remission of symptoms, but still uses eye drops every other day.

MU is used for the treatment of body flaccidity, contouring, and muscle anchoring.^1^ It acts by emitting vibrating waves, which generate molecular friction and heat, creating an area of coagulation. This leads to a tissue healing and retraction process (lifting).^1^, ^2^ According to the vibratory frequency, its energy is concentrated on a certain layer of the superficial musculo-aponeurotic system and promotes the aforementioned process.^1^, ^3^ Because of that, it is important to pay attention to the correct use of the technique, which consists in using the appropriate tip, respecting the pre-established number of discharges for each area and, above all, never pointing the transducer towards the eyes, when it is used on the periocular or facial regions.^2^, ^3^

Similar cases have been previously observed, in which state-of-the-art devices were used and coursed with equivalent alterations, that is, symptoms immediately after the procedure, cataracts in the shape of spicules and difficult-to-control glaucoma. It is noteworthy that the patient had already undergone the same procedure before, with an older device, without complications.

Due to these significant adverse effects, the Brazilian Society of Glaucoma sent a letter in November 2019 to the Brazilian Society of Dermatology, issuing the necessary warning and highlighting the peculiar finding of a “spiky” cataract. It is important to emphasize that ophthalmological complaints after dermatological procedures should be evaluated by an ophthalmologist.^4^, ^5^

The authors consider this communication to be opportune because it serves as a warning to colleagues, who must be very attentive to the use of new technologies. In the present case, specifically, the possible severe ophthalmological complications related to the use of MU.^5^

## Financial support

None declared.

## Authors' contributions

Mariana Marques Rechuan: Drafting and editing of the manuscript.

Jordana Cezar Vaz: Drafting and editing of the manuscript.

Fernanda de Azevedo Pereira Marques: Drafting and editing of the manuscript.

David Rubem Azulay: Drafting and editing of the manuscript; critical review of the manuscript.

## Conflicts of interest

None declared.
